# Challenges in translational drug research in neuropathic and inflammatory pain: the prerequisites for a new paradigm

**DOI:** 10.1007/s00228-017-2301-8

**Published:** 2017-09-11

**Authors:** A. Taneja, O. Della Pasqua, M. Danhof

**Affiliations:** 1 Division of Pharmacology, Leiden Academic Centre for Drug Research, Leiden University, Leiden, The Netherlands; 2 Clinical Pharmacology Modelling & Simulation, GlaxoSmithKline, Uxbridge, UK; 3 Clinical Pharmacology & Therapeutics Group, University College London, London, UK

**Keywords:** Neuropathic pain, Inflammatory pain, Chronic pain, Hyperalgesia, Analgesics, PKPD modelling, Drug development

## Abstract

**Aim:**

Despite an improved understanding of the molecular mechanisms of nociception, existing analgesic drugs remain limited in terms of efficacy in chronic conditions, such as neuropathic pain. Here, we explore the underlying pathophysiological mechanisms of neuropathic and inflammatory pain and discuss the prerequisites and opportunities to reduce attrition and high-failure rate in the development of analgesic drugs.

**Methods:**

A literature search was performed on preclinical and clinical publications aimed at the evaluation of analgesic compounds using MESH terms in PubMed. Publications were selected, which focused on (1) disease mechanisms leading to chronic/neuropathic pain and (2) druggable targets which are currently under evaluation in drug development. Attention was also given to the role of biomarkers and pharmacokinetic-pharmacodynamic modelling.

**Results:**

Multiple mechanisms act concurrently to produce pain, which is a non-specific manifestation of underlying nociceptive pathways. Whereas these manifestations can be divided into neuropathic and inflammatory pain, it is now clear that inflammatory mechanisms are a common trigger for both types of pain. This has implications for drug development, as the assessment of drug effects in experimental models of neuropathic and chronic pain is driven by overt behavioural measures. By contrast, the use of mechanistic biomarkers in inflammatory pain has provided the pharmacological basis for dose selection and evaluation of non-steroidal anti-inflammatory drugs (NSAIDs).

**Conclusion:**

A different paradigm is required for the identification of relevant targets and candidate molecules whereby pain is coupled to the cause of sensorial signal processing dysfunction rather than clinical symptoms. Biomarkers which enable the characterisation of drug binding and target activity are needed for a more robust dose rationale in early clinical development. Such an approach may be facilitated by quantitative clinical pharmacology and evolving technologies in brain imaging, allowing accurate assessment of target engagement, and prediction of treatment effects before embarking on large clinical trials.

## Introduction

Chronic pain remains a debilitating condition with high morbidity and impact on an individual’s quality of life. Currently, marketed analgesic drugs are at best moderately effective, and many of them are known to cause unacceptable side effects or have been linked to long-term safety issues [1, 2]. Despite these limitations and an improved understanding of the molecular mechanisms of nociception [3, 4], research efforts in drug discovery and development continue to rely upon empirical methods; most of which are based on behavioural measures of evoked pain or symptomatic relief.

The implications of the empirical evaluation of novel compounds for pain are illustrated by the incident in the recent trial with BIA-107424 [5, 6], a fatty acid amide hydrolase inhibitor, in which a subject died and five others experienced serious adverse events during dose escalation in healthy subjects. The dose rationale and escalation criteria were primarily guided by overt safety findings rather than data on target engagement, drug exposure (pharmacokinetics) or biomarkers of the pharmacological activity (pharmacodynamics) of the active moiety.

Here, we provide an overview of the key challenges for the development of novel analgesic drugs with special focus on the shortcomings of current experimental protocols and decision criteria for the progression of compounds into clinical trials. In fact, we highlight that evidence of concentration-effect (PKPD) relationships is essential but not sufficient for translation and prediction of treatment response in humans. The dose rationale for analgesic drugs needs to take drug exposure at the site of action, drug binding, and downstream pharmacological effects into account. These principles have been outlined by Vicini *et al.* who proposed a set of general criteria for the progression of compounds into humans and proof of concept studies [7]. Gathering such evidence imposes the use of an integrated approach that provides insight into the interaction between pharmacokinetics, pharmacodynamics, and the underlying nociceptive mechanisms.

### The current landscape for the discovery and development of analgesic drugs

In spite of extensive research on the mechanisms of nociception and pathophysiology of pain, drugs acting on the opioid receptor system or showing non-steroidal anti-inflammatory mechanisms have been the only successful molecules over the last decades, with very few novel selective mechanisms shown to be effective in clinical practice [8–10]. In recent years, pregabalin and duloxetine have been added to the treatment armamentarium. Nevertheless, these treatments have not been able to satisfactorily address the issue of refractoriness to pharmacotherapy [11]. This shortcoming appears to be a consequence of the choice of experimental models of pain in early drug discovery, which are used to screen compounds according to their effect on symptoms, irrespective of the lack of construct validity [12, 13]. Most experimental models in non-clinical species detect drug effects following a noxious stimulus, but the mechanisms of nociception associated with evoked pain involve substrates that are non-specific for the pathophysiology in patients, leading to frequent false positive results. One example of such non-specificity is illustrated by the development of aprepitant, an NK1 antagonist that shows efficacy in preclinical species, but failed in clinical studies [14, 15]. Similarly, clinical data with FAAH inhibitors shows that pain modulation via the CB1 receptor system in humans does not reproduce the findings observed in preclinical models [16].

From a clinical perspective, similar challenges occur as guidelines for diagnosis and treatment rely on evidence of persistent allodynia and/or hyperalgesia that manifest after the onset of changes induced by hypersensitisation and neuroplasticity [11, 17, 18]. Therapeutic interventions at this stage of the disease are likely to be suboptimal since structural and physiological changes that have taken place may be irreversible or cannot be reset by further neuronal remodelling.

**Fig. 1:**
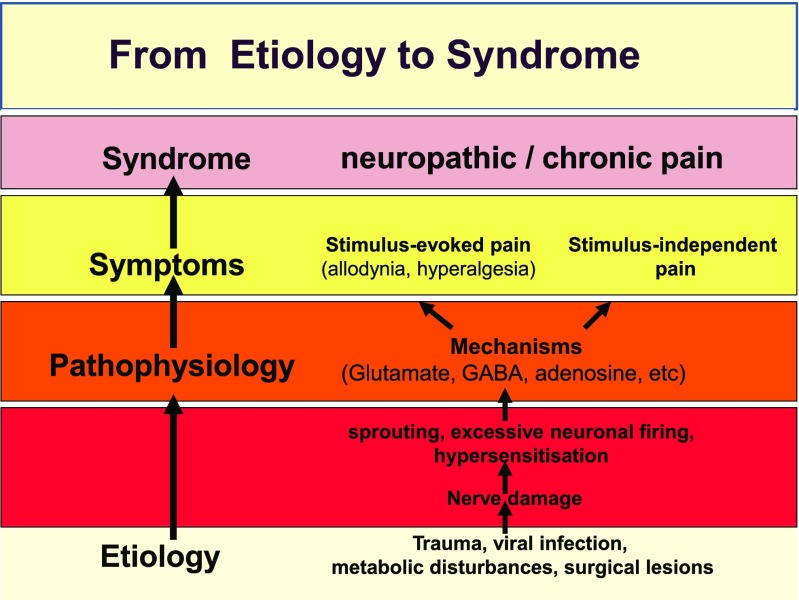
A flow diagram showing the different dimensions and progression from aetiology to the ultimate clinical overt manifestations of neuropathic and chronic pain. The current paradigm for the screening of novel candidate molecules is based on the evaluation of drug effects on overt behavioural symptoms of pain. This represents an important limitation for the identification of efficacious compounds in humans and is partly explained by the lack of (1) diagnostic markers that allow the detection of pathophysiological or structural changes before the onset of overt symptoms and (2) clinical and non-clinical experimental models that reflect the timing and progression of the disease in patients with chronic and neuropathic pain

Given that neuropathic and chronic pain results from a preceding dysfunction in sensory signalling (Fig. 1), the identification of effective treatments requires further insight into the reversibility of the underlying dysfunction as well as the timing of intervention relative to the onset of the disease. Novel therapeutic interventions need to be focused at the dysfunction in signalling pathways rather than primarily on pain relief. Moreover, given that the period between the onset of disease and overt symptoms is associated with irreversible changes in neuronal activity, the timing of any therapeutic intervention becomes a key factor for the success of a treatment. This situation clearly contrasts with inflammatory pain conditions, for which diagnosis is reasonably immediate relative to onset of the underlying dysfunction (i.e. inflammatory reaction), enabling timely interventions. In fact, treatment of acute inflammatory pain following injury is usually efficacious.

#### Pathophysiology of neuropathic and chronic pain

**Fig. 2:**
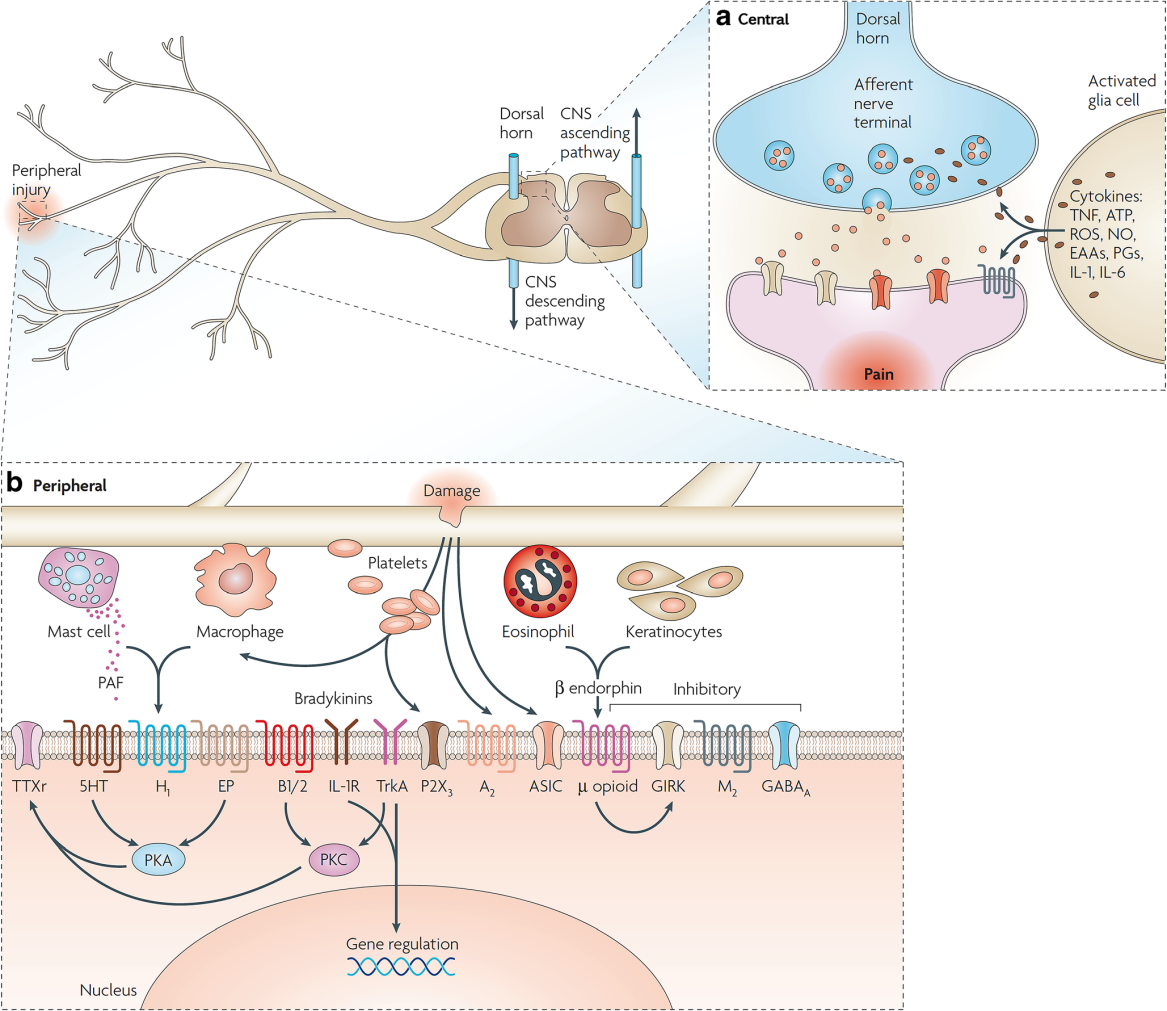
Central and peripheral mediators and neurochemicals associated with the pathophysiology of inflammatory, neuropathic and chronic pain. **a** Upper panel: Following nerve injury, neurochemical modulation of synaptic transmission occurs in the dorsal horn, post-synaptic receptors and ion channels are activated by excitatory amino acids released presynaptically and further sensitised by cytokines from activated glial cells. **b** Lower panel: Peripheral mediators of pain transduction after tissue injury. Following tissue injury, mast cells, macrophages, and other injured cells directly or indirectly release numerous chemicals that alter the sensitivity of receptors and ion channels on peripheral nerve endings. These receptors release secondary messengers such as protein kinase A and C, which can activate other membrane bound receptors and gene transcription. *A*_2_ adenosine 2 receptor, *ASIC* acid sensing channels, *B1/2* bradykinin receptors, *CNS* central nervous system, *EAA* excitatory amino acids, *EP* prostaglandin E receptor, *GABA* γ-amino-butyric acid, *GIRK* G-protein coupled inwardly rectifying K+, *H*_1_ histamine receptor, *5HT* 5-hydroxy-tryptamine, *IL 1/2* interleukins 1/2, *M*_2_ muscarinic-2 receptor, *NO* nitric oxide, *P*_2_ *X*_3_ purinergic receptor X_3_, *PAF* platelet-activating factor, *PGs* prostaglandins, *ROS* reactive oxygen species, *TNF* tumour necrosis factor, *TTXr* tetrodoxin receptor, *TrkA* tyrosine receptor kinase A. Reprinted with permission from [4]

The amplification of a noxious stimulus arising from tissue injury and inflammation involves multiple molecular and cellular pathways, which ultimately contribute to the processing and perception of pain. These pathophysiological changes are schematically depicted in Fig. 2. Following cellular or tissue injury, there is an inflammatory reaction that leads to the release of inflammatory mediators that sensitise sensory receptors on peripheral nerve endings [19, 20]. These receptors are known to release secondary messengers such as protein kinase A and C, which activate other membrane-bound receptors and trigger gene transcription.

Both the peripheral sensitisation and transduction processes described above can develop into central sensitisation, which results from functional and histological changes in the afferent fibres that are present in the dorsal horn of the spinal cord [4]. In the case of neuropathic pain, additionally, there is neuronal hyper-excitability and irregular firing. Sympathetic neuronal sprouting occurs at the cell bodies of afferent neurons in the dorsal root ganglion, which may account for sympathetically mediated pain. Peripheral nerve injury also causes enhanced NMDA activity, glial cell activation, and hypertrophy within the spinal cord. Furthermore, activated microglia expresses purinergic receptor subtypes and releases pro-nociceptive cytokines such as IL1, TNF-α, and neurotrophins which exacerbate nociceptive transmission and ultimately sustain the symptoms of hypersensitisation [21, 22].

**Figure:**
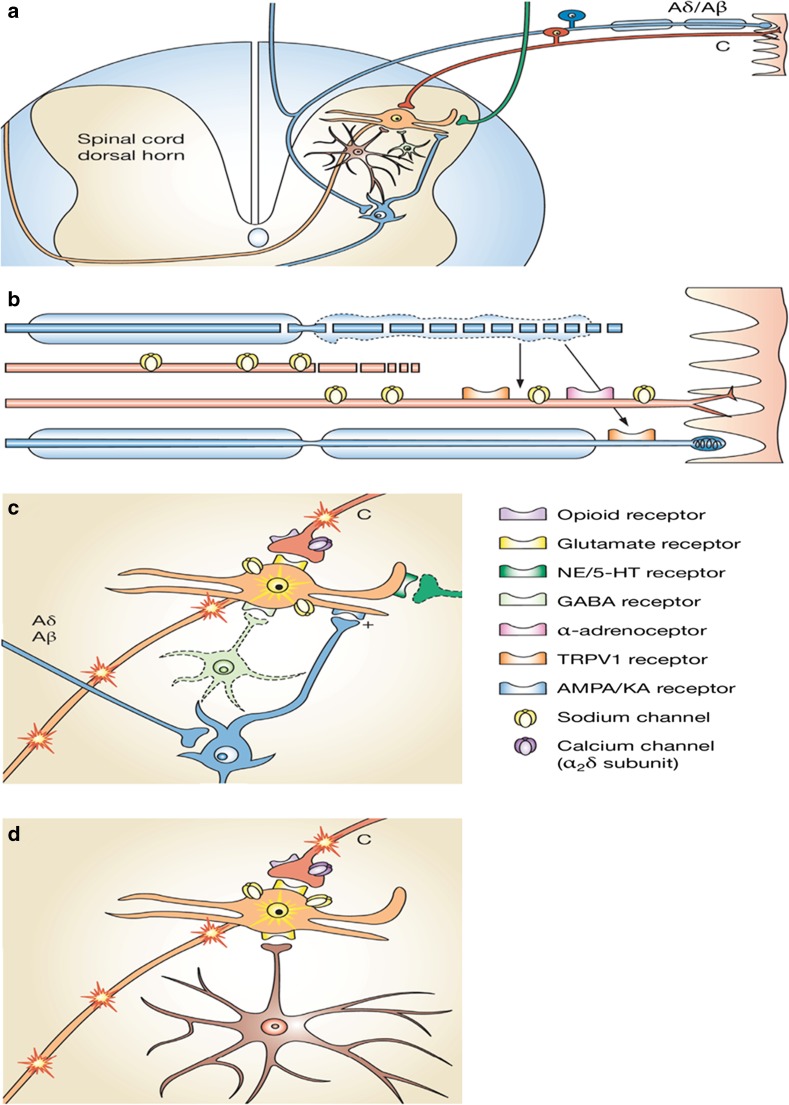

Similarly, peripheral sensitisation, which results from the sensitisation of nociceptors by inflammatory mediators, neurotrophic factors, or pro-inflammatory cytokines, is associated with intense, repeated, or prolonged action potential generation in primary sensory afferents. Such changes are mediated by altered expression and activity of voltage-gated sodium and calcium channels [23, 24]. The activation threshold of nociceptors is lowered and their firing rate increased, leading to symptoms such as allodynia and hyperalgesia. These peripheral processes play an important role in the development and maintenance of central sensitisation [25], which ultimately causes irreversible increased neuronal excitability [26].

While both peripheral and central sensitisation plays a role in chronic pain, central sensitisation is more predominant in neuropathic pain. In fact, not only neurons but also glial cells (e.g. astrocytes and microglia), as well as infiltrating mast cells are involved in the generation and maintenance of central sensitisation [23], which explains why established pain is more difficult to suppress than acute pain [24, 25]. Central sensitisation is also associated with the expansion of dorsal horn neuron receptive fields, reduction in central inhibition, and long-lasting spontaneous dorsal horn neuron activity [23, 27]. Such activity leads to sensory response to low intensity stimuli, reflecting altered neural connections following sprouting of Aß fibres into the superficial laminae. In addition, these changes cause pain signalling to spread to uninjured tissue, i.e. secondary hyperalgesia. This process is known as “wind-up” in that the response of sensitised dorsal horn neurons is exaggerated relative to normal physiological conditions [23, 25]. An overview of the mechanisms of peripheral and central sensitisation is depicted in Fig. 3.

In summary, sensitisation of the nervous system in response to neuropathic and chronic pain results from changes in neuronal structure, connections between neurons, and alterations in the quantity and properties of neurotransmitters, receptors, and ion channels (Table 1). These structural and functional adaptations, i.e. neuroplasticity, cause a shift in the balance between excitatory and inhibitory systems and ultimately in increased pain [19].

**Table 1:** Functional components of neuropathic and chronic pain pathways, key anatomical substrates, and their importance

Process and underlying mechanism	Major neurotransmitter/s (target/tissue)	Time of release/activation	Consequences	Importance/remarks
**Pain signalling/peripheral sensitisation at primary afferent neurons**				
Peripheral nociceptor sensitisation (hyperexcitability)	Substance P (receptors on peripheral terminals and NK1 receptors, plasma membrane of cell bodies, dendrites of non-stimulated neurons) [19, 22, 28–30]	Early in the development of neuropathic pain	Sensitisation of peripheral terminals, increased firing rate.	This mechanism explains hyperalgesia as consequence of hypersensitisation
Activation of purinoceptors on microglia	Purinergic pathways [19, 22]			
Release of excitatory amino acids (EAA)			Induction of neuropathic pain state	
			Release of TNF-α	
Cytokine release following tissue injury is released by macrophages and nerve cell	Cytokines (receptors on blood monocytes)	Early, within 24 h of the onset of inflammatory response	Mediates the inflammatory state	Ectopic hyper-excitability due to increase in nerve cell interaction, resulting in a vicious cycle of inflammation
Inflammation (active macrophage infiltrate)	TNF-α		Activation and release of platelet-derived growth factor (PGDF)	TNF-α is the primary inflammatory mediator involved in certain nerve injuries (e.g. lumbar disc herniation)
Activation of phospholipase A_2_ (PlA_2_) enzyme on cell membranes	Release of arachidonic acid from the cell membrane phospholipid		Increase in prostaglandin concentrations, which in turn increase the production of glutamate	
PlA_2_ activation triggers two competing pathways, i.e. cyclo-oxygenase (COX) and lipo-oxygenase (LOX)	Prostaglandins (peripheral nociceptors, PGE_2_ receptors in smooth muscle)		Sensitisation of peripheral nociceptors, localised pain, hypersensitivity in uninjured tissue	While IL-6 is the primary chemical mediator in pain, IL-10 is a natural anti-inflammatory cytokine. The net inflammatory response is the result from these opposing effects
	Thromboxane (TXA_2_ receptors on platelets)			
	Leukotrienes (receptors on smooth muscle)		Leukotriene-induced platelet activation and constriction of smooth muscle	
Release of interleukins	IL-1β, IL-6, IL-8, IL-10 (peripheral nociceptors) [31]	Within the first few hours of tissue injury	Increased vascular permeability and leukocyte attraction	
			Stimulation of the production of pro-inflammatory mediators such as PGE_2_, COX-2, and matrix metallo-proteases (MMP)	
**Pain processing**				
Central sensitisation (spinal cord)	Glutamate (presynaptic opioid, glutamate receptors)	Unknown	Dynamic mechanical allodynia	Spread of spinal hyper-excitability
	Substance P (calcium channels-(α_2_-δ))		Punctate mechanical allodynia	Expansion of neuronal fields [22, 23, 32]
	Protein kinase C (NMDA receptors) and purinoceptors [22, 23, 32]			
Phenotypical switch	Calcitonin gene-related peptide, substance P (dorsal horn receptors)	Unknown	Input from mechanoreceptor A fibres is perceived as pain (i.e. dynamic and punctuate allodynia)	Increased synaptic transmission, which is considered the most important steps in the development of chronic pain [25]
Nociceptor peptides normally expressed by A δ and C fibres are expressed by large myelinated Aß fibres				
Descending dysinhibition	GABA (GABA receptors)	Late manifestation, months to years after neurological insult [33]	Loss of inhibitory synaptic currents	Selective apoptotic loss of GABAergic neurons in superficial dorsal horn of the spinal cord
	Endogenous opioids (μ receptors)			
Functional degeneration of interspinal inhibitory interneurons	Serotonin/norepinephrine, dopamine (α-2, 5-HT receptors at the dorsal horn inhibitory interneurons)	Protracted several weeks after peripheral nerve injury [1, 26, 34]	Enhanced signal transmission in the dorsal root ganglion	Inhibition or prevention of apoptotic loss leading to functional degeneration could provide disease modifying effect in neuropathic pain
	Glutamate (glutamate receptors, purinergic receptors [22, 25, 34]			
Decreased supraspinal descending modulation				Structures in the mesencephalic reticular formation—possibly the nucleus cuneiformis and the periaqueductal gray area are involved in central sensitisation in neuropathic pain [25]
Descending facilitation				Interestingly, advanced functional MRI (fMRI) techniques show that the same brainstem structures are active in humans with allodynia
**Pain perception/plasticity in the brain**				
Intense and persistent nociceptive input involving limbic circuitry. Long-term down-regulation of dopamine receptors and dopamine production, enhanced glutaminergic transmission from prefrontal cortex to nucleus accumbens [35]	Dopamine, glutamine	Plasticity onset occurs at a late stage; associated with chronicity of pain	Maintain synaptic plasticity.	Similar changes occur in the brain, particularly in the cortex and can be measured experimentally and by functional magnetic resonance Imaging or PET.
			Develop and maintain inflammatory hyperalgesia	Dramatic alterations in cortical spatial maps can be detected after nerve injury that may contribute to phantom pain [26, 35]

#### Pathophysiology of inflammatory pain

**Fig. 4:**
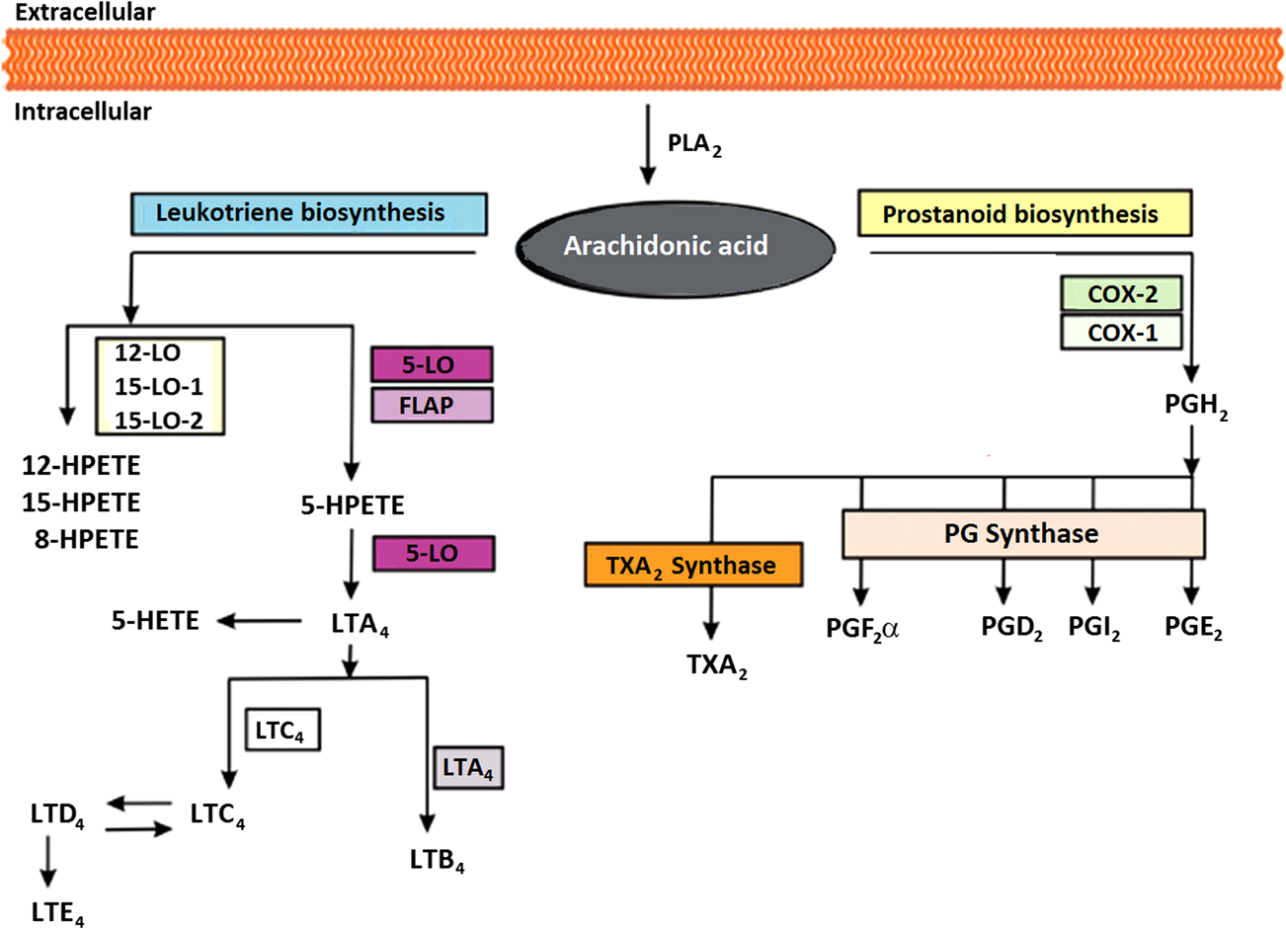
Overview of arachidonic acid cascade associated with inflammatory pain response. Arachidonic acid is released from cellular membranes by cytosolic phospholipase *A*_2_ *(PLA*_*2*_ *)*. The free arachidonic acid can further be converted to eicosanoids by three different pathways involving lipoxygenases (*LO*), cyclooxygenases (*COX*), and the cytochrome P450 monooxygenase pathway (not shown), respectively. COX enzymes catalyse the conversion of arachidonic acid to prostaglandin G2, which is reduced to prostaglandin *H*_*2*_ *(PGH*_*2*_ *)*. By specific prostaglandin (*PG*) and thromboxane *(TXA*_*2*_ *)* synthases, PGH_2_ is subsequently converted to different prostaglandins and thromboxane *A*_*2*_. Different LO enzymes convert the arachidonic acid to biologically active metabolites such as leukotrienes and hydroperoxyeicosatetraenoic acids (*HPETEs*). In the leukotriene pathway, arachidonic acid is converted to 5-HPETE, which is further metabolised to the unstable leukotriene *A*_*4*_ *(LTA*_*4*_ *)*. *LTA*_*4*_ is converted to *LTB*_*2*_ or the cysteinyl-containing *LTC*_*4*_, *LTD*_*4*_, and *LTE*_*4*_. Adapted from [39]

In contrast to neuropathic pain, tissue injury-associated pain typically improves as inflammation resolves. There are instances, however, where the inflammatory/injury state may resolve but a component of pain persists. In inflammatory pain, hypersensitivity is the consequence of alterations in the sensitivity of the nociceptors, activity-dependent changes in the excitability of spinal neurons and phenotypic changes in sensory neurons innervating the inflamed tissue. In brief, tissue injury leads to the release of arachidonic acid and inflammatory mediators, including cyclo-oxygenase 2 (COX-2), tumour necrosis factor (TNF-α), and interleukins (IL-1β, IL-6), which increase the transmission of painful stimuli. Whereas the interplay between different cytokines and inflammatory mediators such as prostaglandins is not fully understood, they also mediate some of the systemic effects of inflammation, such as fever [36–38]. An overview of the inflammatory cascade is shown in Fig. 4. Moreover, the induction of cytokines stimulates the expression of the inducible form of nitric oxide synthase (iNOS), which in turn provokes the release of nitric oxide (NO). In addition to local cellular events, potassium, prostaglandins, bradykinins, ATP, and other mediators from damaged cells trigger the nociceptors to send afferent impulses via the dorsal root ganglion to the spinal cord. Afferent information is then transmitted via second-order neurons in the dorsal horn through the spinothalamic tract to the thalamus and sensory cortex [40].

Undoubtedly, inflammatory pain and neuropathic pain share common mechanisms [41, 42]. It is the time course and relative contribution of each mechanism that seems to differ. Characterisation of such differences is critical to prevent the transition from acute pain to a persistent, chronic state. It becomes evident that novel approaches are needed that not only involve analgesia but also modify the progression of pain as a disease [43–47]. Further details on the pathophysiology of inflammatory versus neuropathic pain can be found elsewhere [47].

### Screening and selection of anti-hyperalgesic compounds

In the next paragraphs, we discuss the weaknesses and opportunities for target selection during the preclinical and clinical evaluation of novel therapeutic strategies for neuropathic and chronic pain, including the prerequisites for the identification of efficacious compounds. These considerations presuppose the implementation of a biomarker-guided approach and integration of quantitative pharmacology concepts as basis for the dose rationale in humans.

#### From hit to leads: target selection

**Fig. 5:**
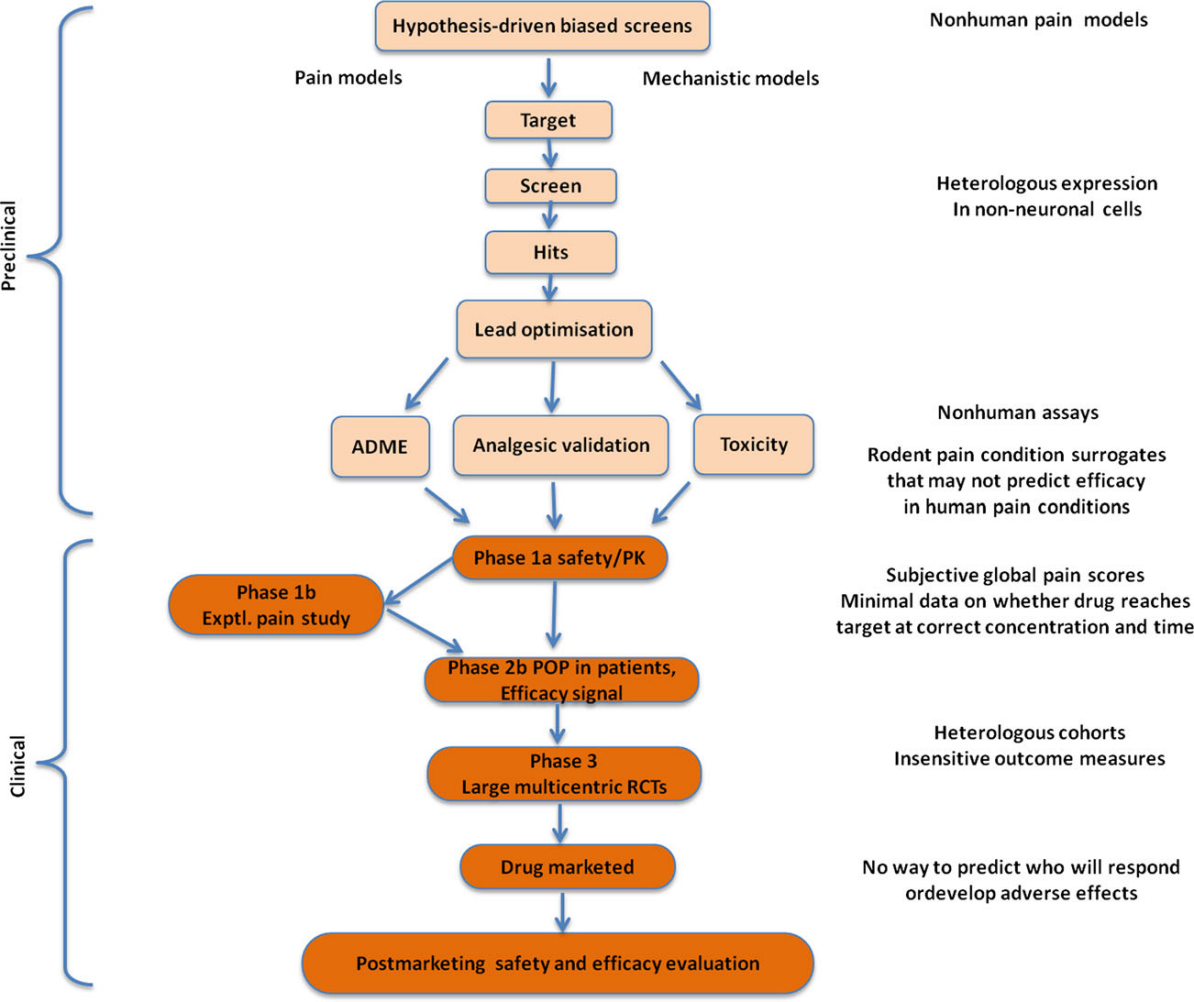
Current paradigm for the discovery and development of analgesic drugs. Typically, R&D efforts start with target selection and end with regulatory approval for the indication in the target patient population. Failures in phases 2 or 3 are a major cause of attrition, and represent the core expenditure in this therapeutic area. Clinical programmes are likely to fail without informative, predictive experimental protocols at the screening phase. The lack of construct validity of preclinical models currently used during drug screening, the irreversibility of changes induced by signalling dysfunction and the absence of early diagnostic tools in humans lead to significant differences in treatment response in animals and humans. Reprinted with permission from [1]

A drug discovery programme begins with target selection, often followed by high-throughput screening and generation of lead compounds. Subsequently, lead optimisation starts based on a set of predefined developability criteria, which are aimed at assessing the drugability of the molecule and its safety profile (Fig. 5) [1]. This approach focuses on the identification of candidate molecules with greater specificity for the target without taking into account the heterogeneity of pain mechanisms or their relative contribution to the progression of the underlying signalling dysfunction. In the case of chronic pain conditions, such a strategy is likely to be flawed, as there may be different targets and/or pathways contributing to the progression of the pathology at different times [48]. Drug discovery efforts in chronic pain will need to consider the lessons from areas such as oncology, where advancements in the treatment of cancer have become tangible not only because of better understanding of the mechanisms of tumorigenesis but also because of a complete redefinition of the diagnostic criteria for patient and treatment selection [49–51]. In this regard, successful therapies are likely to be coupled to early diagnosis and identification of the relevant targets.

#### From behavioural measures to markers of pain signalling: candidate selection

The identification candidate molecules which show potential clinical efficacy in chronic pain conditions will depend on a number of factors. First, drugs should contribute to restoring the underlying signalling dysfunction and promote the reversibility or remodelling of neuronal activity. Evidence should be obtained about the degree or extent of target engagement required to obtain such effects. As these effects precede clinical symptoms, improved diagnostics will have to be developed in parallel to the evaluation of novel compounds. To date, such a scrutiny has never been considered as the basis for the development of analgesic drugs, given that current medicines have been selected based on their effect on behavioural measures of pain. In fact, experimental studies in pain are often considered “behavioural studies,” in which responses to graded-strength mechanical, thermal, or chemical stimuli (nociceptive) are measured. Furthermore, pain measurements are based on the detection of a change in the threshold or response to an applied stimulus, making them unsuitable for the quantification of spontaneous pain, i.e. a major feature of chronic pain conditions in humans [52]. Previously, Huntjens *et al*. have argued that such measures lack the sensitivity and specificity to be able to discriminate between compounds with different pharmacological properties [53]. Also, these measures may not correlate with the time course of the underlying inflammatory and nociceptive response [54]. The authors further argue that behavioural endpoints of pain such as those measured in preclinical models represent a qualitative rather than a quantitative measure of drug effect in vivo, with little correlation with the mechanisms of action [53]. These views are corroborated by Woolf, who has highlighted the fact that while different pain assessment tools have been developed, they are mainly designed to measure pain intensity, not its identity [1].

Although there are a number of potential mediators associated with neuronal firing and hypersensitisation, identification of the pathway(s) determining the progression of disease remains elusive. Consequently, in the absence of easily measurable markers of signalling dysfunction, behavioural measures continue to be the endpoint of choice in the development of analgesic drugs.

#### The lack of predictive value of animal models of pain

The predictive value of any animal model resides in our ability to understand which mechanisms are involved and which endpoints reflect drug effects that can be linked back to these mechanisms, so that one can accurately assess and interpret correlations between pharmacokinetics and pharmacodynamics [13, 55]. Yet, there is no consensus on how well a compound should perform in animal models before it is selected for study in patients [56, 57]. Translational studies in animal models and human subjects have identified an association between pathological mechanisms and symptoms, such as tactile allodynia and central sensitisation. However, it is not clear if this association represents a mechanistic underpinning for this particular symptom. Thus, a causal path analysis is missing to explore if a given endpoint is truly reflective of the mechanisms that are engaged during treatment (e.g. that tactile allodynia is a consequence of central sensitisation) or may also result from other related pathological processes (e.g. tactile allodynia may be caused by sprouting). In this regard, observed behavioural measures such as the reduction of spontaneous activity characteristic of pain as in the formalin-induced pain (FIP) model or the reduction in spontaneous activity by adjuvant (RSAA) model represent an advantage, but yet do not provide evidence on how changes in spontaneous behaviour correlate with the underlying biological substrates [58, 59].

A critique by van Der Worp *et al*. concludes that while animal models have contributed to our understanding of disease mechanisms, in most cases they are not suitable to inform clinical trials. They attribute the translational differences across species to the methodological flaws in preclinical protocols that cause a systematic bias in the evaluation of drug effects [60].

Apart from considerations of how translatable the preclinical models of disease are, findings from these studies are often confounded by poor experimental design. Understandably, practical constraints often preclude the design of such experiments. Yet, the tendency to design low-efficiency experiments should be eschewed. For instance, a common experimental fallacy is the collection of exposure data primarily around the expected *C*_max_ under the misconception that is maximally informative on response [61]. Meta-analyses of over 100 published studies have revealed that random allocation of treatment was done in less than 28% of the studies, while observer blinding was done in less than 2% of these publications. Usually, no formal sample size calculations are performed a priori to determine the appropriate number of animals given the expected effect size. In other cases, unplanned interim analyses are included in the study and experimental protocols continued when interim results are in favour of the working hypothesis. When results show a promising trend, additional data are collected, a practice commonly referred to “sampling to a foregone conclusion” [60].

A related aspect is the design of informative experiments that enable the generation of data which has translational value and/or elucidates the pharmacology of the compound. Gabrielson *et al*. have postulated the concept of quantitative pharmacological reasoning. Preclinical experiments should be designed taking into account exposure-time and exposure-response relationships. It is important to describe the delay in the onset of effect manifested by some compounds relative to the start of the treatment. On the other hand, in certain cases, systemic exposure data may not be informative or reflect tissue or CNS drug levels [62]. This leads to a key concept in drug discovery development, i.e. that of designing studies which provide insight into target engagement. To accomplish this objective, Gabrielson *et al*. propose an integrative approach for which the following three prerequisites should be met: (a) exposure information at the target site is collected, which can be obtained for example by brain microdialysis; (b) target occupancy is quantified by positron emission tomography (PET) imaging; and (c) the pharmacological activity is characterised with the help of mechanism-based biomarkers which allow characterisation of upstream signalling events [61].

#### Shortcomings of challenge models and clinical trials

For compounds that do advance to clinical testing, commonly used experimental models of pain in healthy subjects suffer the same limitations of those used in preclinical species. Based on the available evidence, it is clear that drug effects on chronic pain conditions cannot be sistematically predicted by pain models [63–65]. As shown in Table 2, most methods are based on evoked pain using stimuli that do not fully reflect the neuronal changes associated with the pathophysiology of neuropathic and chronic pain [66, 68]. In addition, dose selection in early human studies is based primarily on empirical criteria, such as the no adverse event level (NOAEL), the human equivalent dose (HED), or the maximum tolerated dose (MTD), without taking into consideration pharmacodynamics or target engagement [69]. The deficiencies arising from these early clinical studies are further amplified in phase II, given that the mechanisms associated with pain in patients may differ considerably from those by which the pain symptoms are induced in animal models of disease or in challenge models of pain in healthy subjects [12]. These differences, together with the lack of early diagnostic tools, are likely to explain most failures in phase II [70]. Moreover, target exposure is overlooked as systemic pharmacokinetics may not reflect drug levels in relevant tissues or organs, and functional imaging or positron emission tomography with radiolabelled ligands is not used in routine clinical research [1, 71].

**Table 2:** Overview of commonly used experimental models of pain in human subjects

Model	Description	Clinical manifestation	Mechanisms	Limitations/application
**Mechanical stimulation models**				
Mechanical stimulation (pinprick, pressure)	Cutaneous stimulation using von Frey filaments, cotton swab, pin-prick, or pressure algometers	Allodynia, pin-prick hyperalgesia	Stimulation of nociceptors and mechanoceptors Aδ and C fibres are stimulated	• Truly noxious stimuli cannot be induced by non-specific cutaneous stimulation • Cutaneous techniques do not mimic nociception • NSAIDs, systemic ketamine, tramadol show analgesic activity [66, 67]
**Chemical-, heat-, or cold-evoked hyperalgesia**				
UVB (ultraviolet B or sunburn)	Hyperalgesia induced by exposing skin area to graded individualised doses of UV B radiation, resulting in dose related erythema	Inflammatory response, allodynia and hyperalgesia Secondary hyperalgesia in erythematous area	Central sensitisation Aδ and C fibres are stimulated	• This model is not sensitive to drugs administered systemically, applied locally or to drug combinations acting via complementary mechanisms of action • NSAID activity has been identified, but no analgesia produced by opioids [66, 68]
Capsaicin-induced pain	Capsaicin is applied topically, intradermally, or intramuscularly Capsaicin exposure leads to acute severe burning pain	Primary or secondary hyperalgesia up to 24 h	Activation of TRPV1 receptor Stimuli mimic the symptoms of hyperalgesia observed in neuropathic pain	• Hyperalgesia is variable as it depends on capsaicin absorption • Opioids, NMDA receptor antagonists, and calcium channel α2-δ ligands attenuate capsaicin-induced hyperalgesia • Limited activity observed with tricyclic anti-depressants and cannabinoids • More C than Aδ fibres activated • Inconsistent results observed during evaluation of drugs with anti-neuropathic pain activity • Lamotrigine and desipramine showed no effects, while gabapentin suppressed hyperalgesia [66–68]
Mustard oil	Model of acute peripheral sensitisation Topical application for a few minutes leads to burning pain followed by an inflammatory reaction in the exposed area	Secondary hyperalgesia and allodynia in surrounding unaffected area	Activation of cation channel TRP amkyrin type I in nociceptive neurons C fibres thought to mediate burning pain A fibres believed to mediate allodynia to light mechanical stimuli	• It has not been widely used in analgesia testing • Limitations similar to those reported with the capsaicin model [66–68]
Thermode burn	Hyperalgesia secondary to first degree burn by exposing healthy subjects to a heat stimulus using a contact thermode	Primary hyperalgesia at the site of exposure, secondary hyperalgesia in adjacent tissue	Central sensitisation Aδ and C fibres are stimulated along with co-activation of Aβ fibres	• NMDA receptor antagonists attenuate mechanical hyperalgesia, but effects are inconsistent with opioids • Intracellular Na channel blockers, opioid receptor antagonists, and purinergic receptor activators [66–68]

**Fig. 6:**
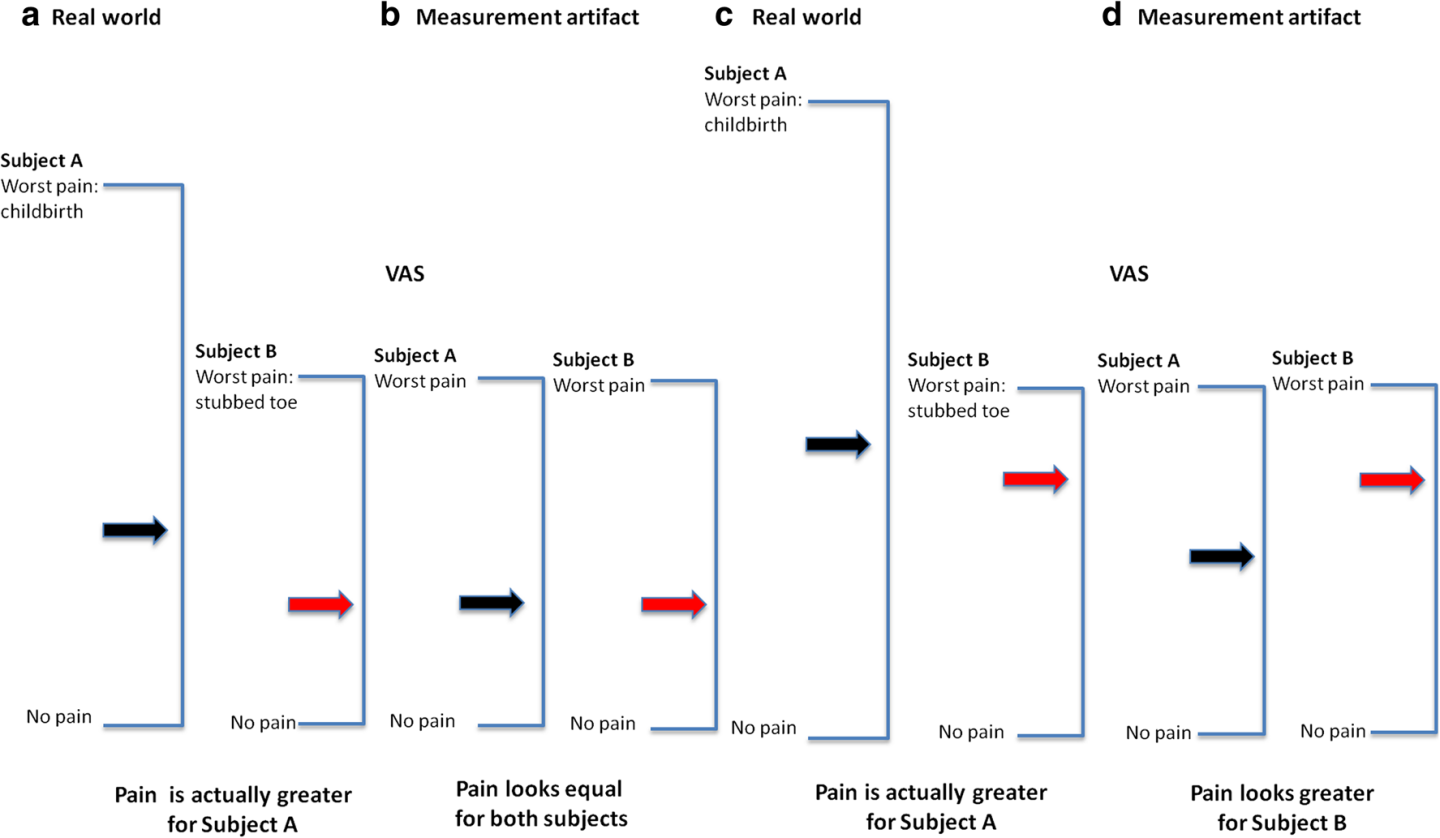
Fallacies of pain comparisons using the visual analogue scale (VAS). If one subject’s worst pain is childbirth and another’s is a stubbed toe, rating the same point on a scale would result in a discrepancy between the actual magnitude of pain experienced and that reported on a conventional VAS. Thus, as depicted in **a**, subject A has experienced greater magnitude of pain than B; it appears that the pain intensity is the same for both subjects. In **c,** the discrepancy is compounded. Subject A experiences pain that is only slightly greater than that of subject B. When maximum pain is treated as it were the same for both subjects, the pain depicted by the *arrows* in **d** erroneously suggests greater pain for B than for A. This is referred to as reversal artefact. Thus, a conventional VAS anchored by “no pain” and “worst pain imaginable” can conceal real differences in pain intensity across subjects. Reprinted with permission from [72]

The assessment of pain symptoms imposes some additional constraints to the evaluation of efficacy above and beyond the fact that the underlying pathophysiological processes may be irreversible. Pain intensity is often measured by a visual analogue scale (VAS), based on a continuous metric ranging from no pain to worst imaginable pain. Moreover, the peak pain sensation for each individual depends on his/her previous experience, which can differ widely. As such, it creates a distortion of the magnitude of the symptoms. As shown in Fig. 6, a standard VAS measurement would equate the maximum pain for all individuals irrespective of their different subjective experience [72, 73]. In analgesic trial reports, it is also customary to report mean outcomes of global pain rating scales, as these studies are based on a hypothesis testing approach [72]. The differences in mean responses of apparently homogenous populations of patients are constructed as evidence of the clinical benefit of the treatment. This is counter-intuitive to the wide interindividual variability intrinsic to chronic pain conditions, which is typically observed in analgesic trials. Subsequently, such a “group” response is used as the basis for dose selection and formal assessment of efficacy in later trials. The lack of attention to interindividual differences and the concept of a “one-dose-fits-all” means that analgesia is achieved in some patients; in others, the same dose could either be ineffective or even toxic. In fact, in many cases, such interindividual variability may be directly caused by differences in the underlying biological substrate [74]. Lee *et al*. showed that variability in gene expression for COX-2 (PTGS2) correlated with pain responses to different analgesics. Subjects homozygous for the gene had a better response to rofecoxib, while the heterozygote responded better to ibuprofen on VAS [75]. Additionally, factors such as gender, ethnicity, age, cultural background, and genetic differences are known to contribute to wide inter- and intraindividual variation in pain response [72, 76]. These covariates not only affect pain perception but also alter the tolerance to painful stimuli.

Interindividual variability in pain response may also be explained by differences in target or even systemic exposure to the drug. The lack of pharmacokinetic sampling and sensitive measures of exposure thwarts most attempts to establish exposure-response relationships [1, 74]. In contrast to situations such as anaesthesia, in which clinical response is closely linked to direct pharmacodynamic measures and to systemic levels of the anaesthetic drug, non-linearity and other time-variant processes in neuropathic and chronic pain make instantaneous circulating concentrations inappropriate metrics of drug exposure. Furthermore, it should be highlighted that the age at which chronic pain occurs also affects its manifestations. While adult nerve injury is characterised by allodynia and hyperalgesia, these symptoms are absent in infants and young children. In this group, nerve injury results in anti-inflammatory response, with unmasking of the pro-inflammatory response around adolescence [77]. This means that standard clinical tests relying on behavioural measures are unlikely to detect the pathology in younger age groups.

In summary, the absence of tools for early diagnosis and the lack of a dose rationale based on target engagement preclude the identification of appropriate targets and compounds capable of restoring or blocking the progression of the underlying signalling dysfunction. The fragmented process used throughout the various phases of development compounds these limitations. Simply, there is little opportunity for the enforcement of the learning and confirming paradigm, which should underpin the rationale for dose selection and progression of a candidate molecule into the late phases of clinical development [78].

### Towards a new paradigm

This review attempts to scrutinise some of the key factors associated high-failure rate in the development of novel analgesic drugs. Notwithstanding a few landmark publications focused on analgesic drug development, thus far, proposed alternative strategies still overlook some of the conceptual elements highlighted in the previous sections of this paper [1, 4, 46, 79]. Our intention is to build on approaches put forth in the aforementioned investigations by identifying a few workable solutions, which can be embedded into the current drug development paradigm.

#### Focus on pathway and target engagement

A shift in the focus of both diagnostic and efficacy measures is required to ensure that treatment is started before the appearance of overt pain symptoms. Consequently, it is necessary to acknowledge the need for preemptive or even prophylactic interventions in which drugs act on relevant pathways associated with hypersensitisation and other structural changes in signalling pathways. This also implies the identification of potentially new targets and pathways; most of which are currently not considered relevant for symptomatic pain relief [80–84].

These principles are in alignment with Morgan *et al.* who suggest that three elements need to be demonstrated for a candidate molecule to survive all phases of development. These are (1) exposure at the target site over a desired period of time; (2) binding to the pharmacological target as expected for its mode of action, and (3) expression of pharmacological activity commensurate with the demonstrated target exposure and target binding [70]. These three elements share some characteristics with the integrative approach previously proposed by other authors working on translational pain research [13, 55, 61]. Of course, evidence of target engagement may not be easily demonstrated in vivo, especially if no overt clinical symptoms are present. Biomarkers and in particular imaging-related biomarkers need to be considered for novel compounds [85]. In addition, in the absence of overt clinical symptoms, correlations must be established between biomarkers and onset of symptoms [86]. Clearly, diagnostic technologies will play a major role, in that target expression or activity will also influence the choice of treatment. From a drug discovery perspective, this implies the co-development of imaging and “wet” biomarkers along with the candidate molecule.

Based on the points highlighted above, it appears that the concept of target engagement might have prevented the incident in the trial with BIA-102474. Irrespective of the mechanisms associated with the serious adverse events observed during the multiple ascending dose study, the rationale for dose escalation was driven by safety thresholds, rather than by pharmacological principles. In fact, dose escalation was progressed without taking pharmacokinetic data into account, despite knowledge about the relatively low potency and poor selectivity of the compound [6].

### The role of biomarkers

Biomarkers can be classified as predictive markers (or markers of pharmacology) and as prognostic markers (or markers of disease/clinical response) [87–89]. In early drug development, the availability of markers of pharmacology can provide evidence of target engagement and consequently activation or inhibition. Such biomarkers can be used as the basis for establishing exposure-response relationships, especially for progression from phase I to phase II studies.

Whereas early diagnosis represent an important challenge, the use of biomarkers is also essential for the dose rationale when the objective of treatment is to prevent the onset of clinical symptoms. In a concept allied to the three pillars of survival, Hargreaves *et al*. have categorised biomarkers into three groups, namely, target, mechanism, and clinical response. According to the authors, biomarkers should be deployed as early as possible first to confirm target engagement, to test whether pathophysiological processes downstream are affected, and subsequently to explore whether a given mechanism affects clinical response [90]. These principles are also reflected in the mechanistic classification proposed by Danhof *et al* [55]. An example of the concept is the presence of KRAS mutation in advanced colorectal cancer, which has been shown to predict the lack of effect of monoclonal antibodies. An immediate application of such a biomarker in oncology is to optimise patient selection, wherein only those patients predicted to benefit most are enrolled into the clinical trial, i.e. in this example patients with HER2/*neu* positive gastric cancer are most likely to respond to trastuzumab therapy [87].

Given the difficulties in identifying the trajectory of response in individual patients, imaging biomarkers may need to be linked to quantitative clinical pharmacology methods. In conjunction with modelling and simulation techniques, imaging and/or wet biomarkers may provide insight into disease processes as well as onset and progression of disease symptoms, discriminating drug from system-specific properties. Such information can be used for inference, extrapolation, and hypothesis generation when evaluating novel molecules or exploring the efficacious dose range.

An inherent difficulty here is to demonstrate that the correlations between biomarker and response are causative and biologically consistent across different stages of disease [91]. Similarly to the use of thromboxane B_2_ and prostaglandins E_2_ as biomarkers for the evaluation of anti-inflammatory drugs acting on the arachidonic acid cascade, functional measures of hypersensitisation and sprouting are required that describe changes in nociceptive pathways. These markers can subsequently serve as a tool for characterising drug effects and establishing correlations between late clinical symptoms (behavioural measures) and early signalling dysfunction.

In this context, Huntjens *et al*. have shown how drug effects on biomarkers unravel differences in the sensitivity of behavioural measures to the selectivity of COX inhibitors [53]. Likewise, we have shown how the exposure-response relationship of prostaglandin E_2_ (PGE_2_), a biomarker of inflammation, can be used to assess target engagement during a phase I study in healthy subjects. This model was used to predict the dose for a future proof-of-concept (PoC) clinical trial. Symptom relief in a subsequent phase IIb study in patients with rheumatoid arthritis was then modelled. The models developed on healthy subject and patient data were then used to simulate the putative correlations between the biomarker (PGE_2_) and the clinical endpoint. Our results indicate that PGE_2_ inhibition correlates with symptomatic improvement, as assessed by core symptom measure. Such a correlation implies the possibility of applying a model-based approach as a means to establish the dose rationale and optimise protocol design for subsequent steps of the clinical programme [74].

In contrast to the advancements observed in the evaluation of anti-inflammatory drugs, potential biomarkers for neuropathic pain, such as glutamate, endocannabinoids, GABA, or cyclo-oxygenase, failed to provide qualitative and quantitative information on the underlying pathophysiological processes [2]. None of these markers appear to satisfy the essential requirements for establishing the validity of a biomarker, namely, i.e. its causal association with the pharmacology and pathophysiology, feasibility, clinical relevance, and ease of use [90]. Notwithstanding this failure, promising results have been observed with functional imaging techniques, such as functional magnetic resonance (fMRI), which allows the identification of different nociceptive phenotypes, and PET, which yields reliable measures of target engagement. In conjunction with challenge models, it may be possible to describe the progression of disease under controlled conditions, such as the induction of secondary allodynia and hyperalgesia following subcutaneous or topical administration of capsaicin [79].

Medical practice will also have to consider early diagnosis and prophylaxis of chronic and neuropathic pain to ensure adoption of a new paradigm for the development of novel, efficacious analgesic drugs. Similar awareness has evolved in the evaluation of drugs for Alzheimer’s disease, where interventions aimed at improving cognitive function are probably unlikely to prevent or mitigate the impact of brain tissue loss, unless treatment is initiated prior to the onset of clinical symptoms [92, 93]. This concept has immediate implications for the development of challenge models. Despite their widespread use in pain research, results from experimental models have translated poorly to clinical analgesia, i.e. experimental protocols and endpoints do not seem to reflect the underlying pharmacological effects of a drug (Table 2). As such, these models do not meet the criteria for early and late biomarkers of disease progression [93–96]. By contrast, Lotsch *et al*. developed a statistical methodology whereby pain models were identified which predicted clinically relevant analgesic drugs [97].

#### Modelling and simulation

A discussion on biomarkers cannot be complete without highlighting their role in model-informed drug discovery and development. The central focus of model-informed drug discovery and development is to use mathematical and statistical models that describe the biological system and drug properties [98]. Hierarchical or population models are among the various approaches currently used. An important property of hierarchical models is the ability to describe variability at individual level by identifying stochastic distributions that describe within and between-subject differences. Subsequently, these models can be used to evaluate the role of distinct components of a biological system as well as to predict treatment effects and disease progression.

**Fig. 7:**
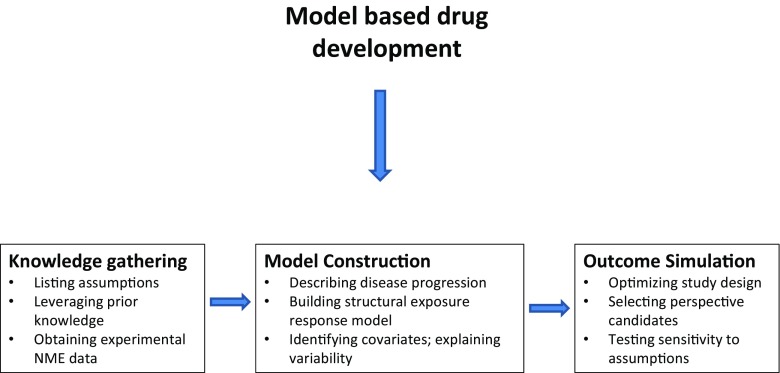
Main steps for the implementation of model-based approaches in drug development. *NME* new molecular entity. Adapted with permission from [99]

Prior to any modelling activities, modelling goals must be clarified; the statistical requirements understood and the most suitable parameterisation identified to ensure that the questions relevant to the modelling exercise are addressed accordingly [78]. This is an iterative process that consists of the following steps: knowledge gathering, parameterisation and model building, parameter estimation, model validation, and prediction or extrapolation by simulation or simulation scenarios (Fig. 7) [99]. At the simplest level of implementation, pharmacokinetic-pharmacodynamic (PKPD) models provide the ability to relate the drug exposure to the time course of the pharmacological effects (or side effects) [100]. Given the role of absorption and distribution processes as well as the presence of functional barriers, pharmacokinetic equilibration models can be incorporated into the analysis to ensure accurate description of drug disposition properties, enabling the characterisation of drug exposure at the biophase (target site). Furthermore, models allow correlations to be established when non-linear processes are required to describe signal transduction or disease progression, both of which are associated with delays between the onset of the pharmacological effect and the time course of drug concentrations. One of the major advantages of a model-based approach is the opportunity to leverage prior information by integrating historical with current data. Existing scientific knowledge may be incorporated in the analysis of experimental data through deterministic or stochastic parameters (e.g. informative prior probability distributions) [99].

Pertinent to the utilisation of biomarkers in drug development is the role of mechanism-based PKPD models, which contain specific expressions to characterise in a strictly quantitative manner, processes on the causal path between drug administration and effect. This includes distribution to the target site, interaction with and activation of the target, transduction, and influence of in vivo homeostatic feedback mechanisms [101]. Mechanism-based models facilitate the integration of information, including pooling of data from different experimental conditions. Using the appropriate parameterisation, it is possible to distinguish drug- from disease-specific properties, as well as to evaluate the impact of influential covariates on pharmacokinetics, pharmacodynamics, and disease.

While hierarchical models provide vital clues on biological variability and on the underlying biology/pharmacology, they may not provide an adequate basis for translation, be it across species or from healthy volunteers to patients. Integration of systems pharmacology with mechanism-based modelling is more likely to provide this translational link [7]. Another important dimension of model-based approaches is the use of models as a design and optimisation tool [102], but these principles are not applied to the development of analgesic drugs. The availability of a validated PKPD model allows for further optimisation of experimental protocols, including the investigation of a range of design characteristics on the power to detect a given effect prior to exposing patients to an experimental drug [103, 104]. In a field where most clinical trials have a conservative design, clinical trial simulations (CTS) offer a unique opportunity to evaluate innovative designs.

In general, CTS utilises two types of models. First, a drug-action (PKPD) model is considered, which comprises pharmacokinetic and pharmacodynamic factors. In chronic diseases, the model also accounts for disease progression. Unfortunately, the lack of knowledge about the mechanisms underlying treatment response in many therapeutic indications has prevented the development of mechanistic PKPD models. Secondly, CTS requires a trial execution model. These models simulate other important aspects of the trial, such as dropout and protocol deviations. Thereby, one can determine all possible outcomes under candidate trial designs. It is also important to stress that CTS allows the investigation of factors that cannot be scrutinised by meta-analysis or empirical design. First, designs that have not been implemented cannot be included in a meta-analysis. Second, it is difficult to separate the influence of multiple design factors, whereas CTS allows the evaluation of a single factor at a time.

The use of such a virtual or statistical experiment allows the assessment of the “trial performance” and as such potential limitations in study and protocol design prior to its implementation [105]. Regrettably, PKPD modelling and CTS have been applied only sporadically in pain research. Data in the published literature suggest that such efforts were made to answer specific research questions, rather than used as the basis for a new drug development strategy [106].

## Conclusions

There are several methodological issues that hinder the development of novel medicines for the treatment of neuropathic and chronic pain. Essentially, these issues arise from the lack of appropriate, early diagnostic criteria, and poor characterisation of the disease dynamics. Multiple molecular and cellular mechanisms act concurrently to produce pain symptoms, which in turn are non-specific manifestations of the underlying nociceptive mechanisms. Most pain research has focused on transient behavioural models of pain that do not necessarily reflect what occurs in a chronic pain patient. A new paradigm is required for the identification of relevant targets and candidate molecules in which pain is coupled to the cause of sensorial signalling dysfunction rather than to the symptoms. In this paradigm, focus should be given to the identification drug targets and candidate molecules that act *before* clinical symptoms evolve, i.e. the assessment of efficacy, or lack thereof, is based on the assumption of disease-modifying properties. Moreover, we envisage the development of a biomarker-guided approach, in which target engagement is used as the basis for dose selection. Biomarkers can be integrated in a systematic manner by PKPD modelling, providing a mechanistic underpinning for the translation of drug effects in preclinical species and prediction of the therapeutic doses in patients.
